# The effect of protein or amino acid provision on immobilization‐induced muscle atrophy in healthy adults: A systematic review and meta‐analysis

**DOI:** 10.1113/EP090434

**Published:** 2024-02-29

**Authors:** Alix K. Hughes, Thomas Francis, Jessica Rooney, Ross Pollock, Oliver C. Witard

**Affiliations:** ^1^ Centre for Human and Applied Physiological Sciences King's College London London UK

**Keywords:** muscle atrophy, muscle disuse, muscle mass, nutrition, protein

## Abstract

Bed rest and limb immobilization are models of muscle disuse associated with skeletal muscle atrophy and reduced strength. The purpose of this systematic review was to examine the impact of protein or amino acid provision before and/or during a period of muscle disuse on muscle atrophy (primary outcome), strength and muscle protein synthesis (secondary outcomes) following a disuse period. We performed a systematic review of Embase, MEDLINE, Web of Science, PubMed and Clinical Trials in December 2022. Eligible studies were randomized controlled trials that combined a dietary protein or amino acid intervention versus control during an experimental model of disuse (bed rest or unilateral limb immobilization) in healthy individuals aged ≥18 years. Nine articles from eight independent trials were identified and rated for risk of bias by two authors. A meta‐analysis of muscle mass data revealed no effect (standardized mean difference: 0.2; 95% confidence interval: −0.18 to 0.57, *P* = 0.31) of protein/amino acid intervention in preventing disuse‐induced muscle atrophy. Although the meta‐analysis was not conducted on strength or muscle protein synthesis data, there was insufficient evidence in the reviewed articles to support the use of protein/amino acid provision in mitigating the disuse‐induced decline in either outcome measurement. Additional high‐quality studies, including the reporting of randomization procedures and blinding procedures and the provision of statistical analysis plans, might be required to determine whether protein or amino acid provision serves as an effective strategy to attenuate muscle atrophy during periods of disuse.

## Abstract (highlights)

- **What is the topic of this review?**
  This is a systematic review of the potential for protein or amino acid provision as an intervention for mitigating skeletal muscle atrophy associated with periods of disuse.
- **What advances does it highlight?**
  Protein or amino acid provision before or during a period of disuse does not prevent the reductions in skeletal muscle mass, strength or muscle protein synthesis associated with immobilization or bed rest, hence future studies are required to investigate effective nutritional interventions during clinical situations of muscle disuse.

## 1 INTRODUCTION

Skeletal muscle atrophy is a physiological consequence of muscle disuse that has clinical relevance to both healthy and clinical populations across the lifespan (Paddon‐Jones et al., 2006; Wall et al., 2015). Muscle disuse results from various, often unavoidable situations, including convalescence during prolonged inclement weather and hospitalization or bed rest owing to injury or illness. Even short periods of muscle disuse lead to significant decrements in muscle mass, as evidenced by a 1.7% decline in muscle volume after 2 days of unilateral limb immobilization that continued to 6.7% total lost over 7 days in 13 young men (Kilroe et al., 2020). More protracted or frequent periods of disuse exacerbate the age‐related decline in muscle mass and function. For instance, 2 weeks of a reduced daily step count (<1000) resulted in a decline of bone and fat‐free mass, muscle function, impaired stimulation of muscle protein synthesis (MPS) in response to ingested protein, and metabolic perturbations in young (Shad et al., 2019) and older (Breen et al., 2013; Devries et al., 2015; Saoi et al., 2019) adults. Moreover, hospitalized older adults were shown to lose muscle mass, which failed to recover to pre‐surgery levels 3 months after discharge (Aarden et al., 2021; Gil et al., 2023). Crucially, the muscle atrophy and subsequent decline in strength following muscle disuse are associated with the development of chronic diseases, including insulin resistance and increased risk of all‐cause mortality (Dirks et al., 2016; Li et al., 2018), and can lead to a reduced quality of life, decreased mobility, and reduced independence, particularly in older adult populations (dos Santos et al., 2017). Hence, establishing targeted and evidence‐based interventions to mitigate muscle atrophy following muscle disuse is crucial for improving muscle health across the lifespan.

Several experimental models have been used to advance scientific understanding regarding the physiological perturbations associated with short‐term periods of muscle disuse. For instance, within a ‘voluntary’ preclinical setting, unilateral leg immobilization in a cast or brace has been used in healthy volunteers as an ‘uncomplicated’ model of disuse, i.e., in the absence of inflammation and/or other metabolic complications. Comparable ‘involuntary’ clinical models of muscle disuse have recruited patients scheduled to undergo musculoskeletal surgery (Dreyer et al., 2018, 2013). However, the scientific control of these clinical trials is often compromised by the inherent individual variation in injury or disease severity between patients and the variable impact of different types of injuries on the magnitude of muscle loss. For example, intensive care unit patients experienced a 17.7% decline in rectus femoris cross‐sectional area (CSA) after 10 days in the intensive care unit, equivalent to 1.77% muscle loss per day (Puthucheary et al., 2013), whereas patients who underwent total knee replacement surgery experienced an 18.4% decline in quadriceps muscle volume over 6 weeks, equivalent to 0.44% muscle loss per day (Dreyer et al., 2013). Hence, the preponderance of studies have used preclinical ‘uncomplicated’ experimental models to investigate the physiological responses to muscle disuse.

Skeletal muscle mass is regulated by the continuous and concurrent stimulation of MPS and muscle protein breakdown. The general consensus exists that a suppressed response of MPS, rather than upregulation of muscle protein breakdown, serves as the primary metabolic driver of muscle atrophy during periods of disuse (Kilroe et al., 2020; Pavis et al., 2023). In this regard, muscle disuse has been shown to elicit a state of anabolic resistance, defined as the impaired response of MPS to ingested protein or amino acid provision (Glover et al., 2008; Wall et al., 2013). Accordingly, previous studies have reported a 36% decline in myofibrillar MPS during 7 days of limb immobilization (Kilroe et al., 2020) and a 50% decline in mixed MPS rates during 14 days of bed rest (Ferrando et al., 1996) in healthy young men, with no effect of short‐term disuse on postabsorptive or postprandial rates of muscle protein breakdown (Pavis et al., 2023). It follows that a suppressed postprandial response of MPS leads to an aggregate catabolic state of negative muscle protein, whereby rates of muscle protein breakdown exceed MPS over a given period of time.

The extracellular availability of amino acids serves as a potent stimulus for MPS (Biolo et al., 1997; Bohé et al., 2001; Svanberg et al., 1996). In this regard, mechanistic studies conducted in resting conditions have demonstrated a ∼3‐fold, albeit transient (3–4 h), increase in postprandial MPS rates that are saturated at 20–25 g (Witard et al., 2014) or 30–40 g (Yang et al., 2012) doses of ingested protein in young and older adults, respectively, with comparable MPS rates observed with the ingestion of equivalent doses of essential amino acids (Atherton et al., 2010; Cuthbertson et al., 2006). Within the context of muscle disuse, combining essential amino acid supplementation and resistance exercise training during a period of bed rest was shown to mitigate ∼60% of the atrophy that was observed with essential amino acid supplementation alone (Brooks et al., 2008), thus highlighting the synergistic effect of exercise and nutrition in modulating MPS (Biolo et al., 1997). Hence, nutritional strategies (with or without a prior exercise stimulus) targeted at increasing amino acid availability and upregulating the stimulation of MPS are of clinical relevance in mitigating the magnitude of muscle atrophy experienced with disuse (Wall & van Loon, 2013; Wall et al., 2015).

Although attempts have been made to investigate the efficacy of nutritional interventions to mitigate disuse atrophy (Ye et al., 2023), no systematic review of protein or amino acid interventions per se has been conducted. Moreover, studies have used variable protein‐based interventions and different models of muscle disuse over a range (from 3 days to 2 weeks) of time frames. Hence, the heterogeneity in studies makes it difficult to discern the efficacy of protein or amino acid provision before or during a period of immobilization or bed rest to mitigate muscle disuse atrophy. Accordingly, a focused systematic review and meta‐analysis of independent nutritional interventions is warranted to devise specific, evidence‐based dietary protein recommendations to ameliorate disuse‐induced muscle atrophy. The main aim of this systematic review and meta‐analysis of randomized controlled trials (RCTs) was to investigate the effect of protein or amino acid interventions before and/or during a period of ‘uncomplicated’ disuse on changes in muscle mass, as measured by a change in size or volume of a muscle or limb region of interest in healthy young and older adults. A secondary aim was to investigate the effect of protein or amino acid interventions on changes in muscle strength and MPS during a disuse period.

## 2 METHODS

The protocol for this systematic review and meta‐analysis was pre‐registered on PROSPERO (CRD42022381687).

### 2.1 Search strategy

A literature search for RCTs designed to investigate the effect of protein or amino acid provision before and/or during a period of bed rest or unilateral limb immobilization on muscle mass in healthy adults was conducted by searching four online literature databases and one clinical trials database. One investigator (A.K.H.) performed a database search of Embase (last search date 6 December 2022), MEDLINE (last search date 7 December 2022), Web of Science (last search date 7 December 2022) and PubMed (last search date 8 December 2022), with the following predetermined search strategy: ((diet* OR supplement* OR administ* OR provision OR ingest*) AND (protein OR amino acid* OR AA OR HMB OR Beta‐hydroxy Beta‐methylbut* OR β‐hydroxy β‐methylbut*)) AND (immobili* OR bed*rest OR dry water OR cast* OR brace). In addition, the Clinical Trials database (www.clinicaltrials.gov) was searched (last search date 13 December 2022) for relevant ongoing or completed immobilization studies. The search strategy used for each database is displayed in the supporting data file. Only human studies were included in the review, and no date or language restrictions were applied to the search. Any conference abstracts that were returned during searches were checked for associated fully published papers. If no additional article could be retrieved, conference abstracts were excluded from the final pool of reports. Reference lists of included studies were reviewed manually for additional studies not identified in the electronic search.

### 2.2 Study selection

The titles and abstracts of studies identified from the search were screened by one investigator (A.K.H.) after removal of duplicates and ineligible records. Thereafter, following abstract screening, the methods sections of potentially eligible studies were screened by the same investigator (A.K.H.). The inclusion and exclusion criteria for the review are outlined in Table 1.

**TABLE 1:** Inclusion and exclusion criteria for study evaluation.

Criterion	Inclusion	Exclusion
Study design	Randomized controlled trial	Not randomized controlled trial
Population	Healthy adults, >18 years old	Children, adults with any comorbidities, animals
Intervention	Any protein or amino acid provision (supplementation or dietary intervention) provided before and/or during a period of immobilization	Non‐protein or amino acid supplementation, e.g., omega‐3, hydroxymethylbutyrate
Comparator	Control group	No control group
Outcome measure	Muscle mass measured by validated means	No measurement of muscle mass

The following information was extracted by one investigator (A.K.H.): (1) author and study date; (2) study type; (3) supplement or dietary intervention; (4) participant age, sex and body mass; (5) disuse model and duration of disuse period; (6) method used for the measurement of muscle mass; (7) the magnitude of change in muscle mass between start and end of disuse period; (8) measurements of muscle strength and/or MPS (if measured); and (9) evidence of prevention of atrophy (determined by significance of muscle mass findings between control and intervention group post‐immobilization) and the key conclusion.

### 2.3 Risk‐of‐bias assessment

A risk‐of‐bias assessment was conducted by two independent investigators (A.K.H. and T.F.) according to the Cochrane risk‐of‐bias tool for randomized trials (RoB 2) (Sterne et al., 2019). Studies were assessed based on assignment to intervention, the ‘intention‐to‐treat effect’, with domains assessing randomization process, deviations from intended interventions, missing outcome data, measurement of the outcome, and selection of reported results. Disagreements between the two researchers (A.K.H. and T.F.) were settled by discussion. Where no consensus could be reached, two additional investigators (O.C.W. and J.R.) were available to arbitrate.

### 2.4 Data synthesis and statistical analysis

The primary outcome measurement was the percentage change in skeletal muscle mass between baseline and the end of the immobilization period. Secondary outcomes included muscle strength and direct or indirect assessments of MPS in response to protein or amino acid provision before or during muscle disuse. Datasets presented in graphical format only were extracted manually using a ruler. If data were missing for muscle mass or if the ability to calculate the percentage change in muscle mass was not possible from the dataset provided, the corresponding author was contacted to obtain the missing dataset required. All contacted authors provided the dataset from the study, and one author indicated a shared control group for two of the studies (Arentson‐Lantz et al., 2020, 2019). Accordingly, the shared group was reorganized such that each paper was compared with a control group consisting of four and five participants, respectively, with the same mean and SD for each group (for details, see Figure 3). Where studies used multiple methods to assess muscle mass, data were reported as the percentage change, with the order of preference as follows: (1) measurements of muscle volume (measured by MRI); (2) muscle CSA (measured by CT scan); (3) leg lean mass; and (4) whole‐body lean mass [measured by dual‐energy X‐ray absorptiometry (DXA)]. This order was selected because measurements of muscle volume and CSA by MRI and CT scans are considered the gold‐standard methods for assessing muscle mass (Pons et al., 2018).

A meta‐analysis was performed on extracted data using a random‐effects model in Review Manager v.5.3. If studies included multiple time points, data from the end of the immobilization period were used in the overall analysis. Given that multiple methods were used to assess muscle mass across different studies, standardized mean difference (SMD) with 95% confidence intervals (CI) was used to express effect size estimates (Deeks et al., 2022). In accordance with Cohen (1988), SMDs were defined as the following: 0.2, small; 0.5, medium; and 0.8, large. The interpretation of findings is complicated by the fact that SMD expresses findings in standard units rather than original units. Accordingly, the Cochrane Handbook recommends the back‐transformation of SMD values into units of a familiar instrument used in studies of the meta‐analysis (Scholten et al., 1999; Schünemann et al., 2023). As such, intervention studies included in the present meta‐analysis that expressed muscle mass or volume as standard deviations at baseline for protein and control groups were used to convert SMD to centimetres cubed (Kilroe et al., 2021) (quadriceps muscle volume) or kilograms (leg lean mass) (English et al., 2016). The following equation was used for both conversions: Back‐transformed mean difference = SMD × SD, where SMD is the standardized mean difference, and SD is the estimated standard deviation of the instrument of choice.

Given the variation in data expression across studies, the percentage change in muscle mass from baseline to post‐immobilization was calculated for all available datasets. Standard error values were converted to standard deviation values where applicable using the following equation: $\mathrm{SD}=\mathrm{SE}\sqrt{n}$, where SD is the standard deviation, SE is the standard error, and *n* is the sample size of the study.

Heterogeneity of results was determined by *I* ^2^. Values of 0%–40% were considered as ‘may not be important’, 30%–60% as ‘may represent moderate heterogeneity’, 50%–90% as ‘may represent substantial heterogeneity’, and 75%–100% as ‘considerable heterogeneity’, as outlined in the Cochrane Handbook (Deeks et al., 2022).

## 3 RESULTS

### 3.1 Study selection

**FIGURE 1:**
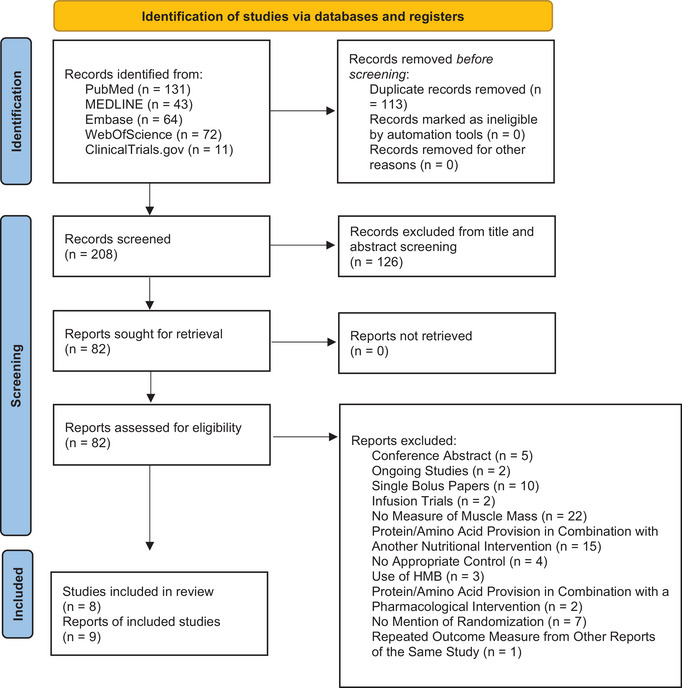
PRISMA flow diagram of the study selection process.

The search revealed 208 potential papers after screening for repeats. This tally was reduced to 82 after title and abstract screening was completed (Figure 1). Of these papers, nine articles met the inclusion and exclusion criteria outlined in Table 1 after fulltext screening.

### 3.2 Study characteristics

The nine articles included consisted of eight independent trials. An overview of the study characteristics, interventions and findings is displayed in Table 2. In total, these studies included 189 participants (171 male and 18 female), all of whom were healthy and with no diagnosed disease state that could accelerate muscle disuse. Six authors were contacted for additional data, all of whom responded. Four of these studies used leucine supplementation (Arentson‐Lantz et al., 2020; Backx et al., 2018; Edwards et al., 2020; English et al., 2016), two whey or dairy supplementation (Arentson‐Lantz et al., 2019; Mitchell et al., 2018), one a whey supplement enriched with leucine (Dirks et al., 2014), one a novel amino acid blend (Holloway et al., 2019), and one a high‐protein diet (Kilroe et al., 2021). The duration of supplement or dietary intervention implemented during muscle disuse ranged from 3 to 14 days (Table 2). Three of the nine studies used bed rest as the mode of disuse, and six used unilateral limb immobilizations, two by full‐leg cast and four by knee brace (Table 2).

**TABLE 2:** Summary of all studies included in the review.

Reference	Study design	Participant characteristics	Disuse mode	Intervention	Assessment of muscle mass	Primary findings
Arentson‐Lantz et al. (2019)	Randomized, single‐blinded, controlled trial	Intervention: Male (*n* = 5), female (*n* = 5), 69 ± 1 years old^a^ Control (shared with above): Male (*n* = 7), female (*n* = 3), 68 ± 2 years old^a^	Bed rest (7 days)	3 days pre‐bed rest and 7 days during bed rest period Intervention: WHEY, i.e. protein from whey protein isolate (0.90 ± 0.01 g protein/kg body mass/day) Control: MIXED diet, i.e. protein from whole‐food, plant and animal sources (0.97 ± 0.01 g protein/kg body mass/day)	Whole‐body and leg lean mass using DXA	No significant difference between groups in changes in whole‐body lean mass, leg lean mass or unilateral knee peak torque
Arentson‐Lantz et al. (2020)	Randomized, double‐blinded, placebo‐controlled trial	Intervention: Male (*n* = 7), female (*n* = 3), 68 ± 1 years old^a^ Control: Male (*n* = 7), female (*n* = 3), 68 ± 2 years old^1^	Bed rest (7 days)	7 days during bed rest period. Intervention: Leucine supplementation (0.06 g/kg/meal) Control: Alanine supplementation (0.06 g/kg/meal)	Whole‐body and leg lean mass using DXA	Significant reduction in leg lean mass loss in leucine group, although no significant difference between groups in changes in whole‐body lean mass
Backx et al. (2018)	Randomized controlled trial	Intervention: Male (*n* = 15), 21 ± 1 years old^a^ Control: Male (*n* = 15), 23 ± 1 years old^a^	Full‐leg cast (7 days)	7 days during limb immobilization. Intervention: 7.5 g free crystalline leucine (2.5 g with each meal) Control: 7.5 g maltodextrin + 7.5 g dextrose monohydrate	Whole‐thigh and quadriceps CSA using single‐slice CT at mid‐thigh	No significant difference between groups in whole‐thigh or quadriceps CSA or in leg extension 1RM
Dirks et al. (2014)	Randomized controlled trial	Intervention: Male (*n* = 11), 68 ± 1 years old^a^ Control: Male (*n* = 12), 70 ± 1 years old^a^	Full‐leg cast (5 days)	5 days during limb immobilization. Intervention: Twice daily high whey protein leucine‐enriched drink (21 g protein per drink) Control: No supplement	Whole‐body and leg lean mass using DXA Whole‐thigh and musculus quadriceps femoris CSA, using single‐slice CT 15 cm proximal to the top of the patella	No significant difference between groups in changes in whole‐thigh or quadriceps femoris muscle CSA or single leg extension 1RM
Edwards et al. (2020)	Randomized, double‐blinded, parallel‐group, placebo‐controlled trial	Intervention: Male (*n* = 8), 22 ± 1 years old^a^ Control: Male (*n* = 8), 23 ± 1 years old^a^	Knee brace, 30° knee flexion (7 days)	7 days during limb immobilization. Intervention: 15 g leucine per day Control: 15 g non‐essential amino acids per day	Leg fat‐free mass using DXA	No significant difference between groups in change in leg fat‐free mass, isometric knee‐extensor strength or isokinetic knee‐extensor strength
English et al. (2016)	Randomized, double‐blinded, placebo‐controlled trial	Intervention: Male (*n* = 6), female (*n* = 4), 51 ± 1 years old^a^ Control: Male (*n* = 6), female (*n* = 3), 52 ± 1 years old^a^	Bed rest (14 days)	14 days during bed rest. Intervention: Leucine supplementation (0.06 g/kg/meal) Control: Alanine supplementation (0.06 g/kg/meal)	Whole‐body and leg lean mass using DXA	Significantly less whole‐body lean mass loss in leucine group mid‐way (7 days) through bed rest; no significant difference between groups in change in whole‐body lean mass at the end of bed rest. No significant difference between groups in postabsorptive or postprandial MPS. Significantly less reduction in knee‐extensor torque at 60 and 180°/s, knee‐extensor work at 180°/s and muscle quality measurements in leucine group vs. control group
Holloway et al. (2019)	Randomized, double‐blinded, parallel‐group, placebo‐controlled pilot trial	Intervention: Male (*n* = 10) Control: Male (*n* = 9) ‘Young’ men, but age not reported	Knee brace, 40° knee flexion (7 days)	7 days pre‐immobilization, 7 days during immobilization. Intervention: novel amino acid blend (AXA2678) containing leucine, isoleucine, valine, arginine, glutamine, lysine, histidine, phenylalanine, threonine and *N*‐acetylcysteine, 23.7 g three times per day Control: maltodextrin, 23.7 g three times per day	Quadriceps muscle volume and CSA using MRI	No significant loss of peak muscle volume or CSA in AXA2678 group, but significant loss in control group
Kilroe et al. (2021)	Randomized, single‐blinded, parallel‐group, controlled trial	Intervention: Male (*n* = 11), 22 ± 1 years old^a^ Control: Male (*n* = 11), 20 ± 1 years old^a^	Knee brace, 40° knee flexion (3 days)	3 days during immobilization. Intervention: High‐protein diet (1.6 ± 0.1 g protein/kg body mass/day during immobilization) Control: No‐protein diet (0.14 ± 0.1 g protein/kg body mass/day during immobilization)	Quadriceps muscle volume using MRI	No significant difference between groups in change in quadriceps muscle volume, quadriceps maximal isometric, concentric or eccentric strength, hamstring concentric strength or myofibrillar MPS
Mitchell et al. (2018)	Randomized, double‐blinded, parallel‐group, placebo‐controlled trial	Intervention: Male (*n* = 15), 51.5 ± 3.8 years old^b^ Control: Male (*n* = 15), 48.5 ± 2.4 years old^b^	Knee brace, 60° knee flexion (14 days)	14 days during immobilization. Intervention: 20 g milk protein isolate once daily (80% casein, 20% whey) Control: maltodextrin once daily, isoenergetic to the intervention supplement	Leg lean mass using DXA Muscle CSA at 20% and 50% of femur length and at 66% of tibia length using pQCT	No significant difference in changes in leg lean mass between groups; no significant difference in myofibrillar MPS or mitochondrial MPS between groups; no group effects seen in knee‐extensor torque, knee‐flexor torque or plantar‐flexor torque

^a^ Mean ± SEM.

^b^ Mean ± SD.

Abbreviations: CSA, cross‐sectional area; DXA, dual‐energy X‐ray absorptiometry; MPS, muscle protein synthesis; pQCT, peripheral quantitative computed tomography; 1RM, one‐repetition maximum.

### 3.3 Risk of bias

**FIGURE 2:**
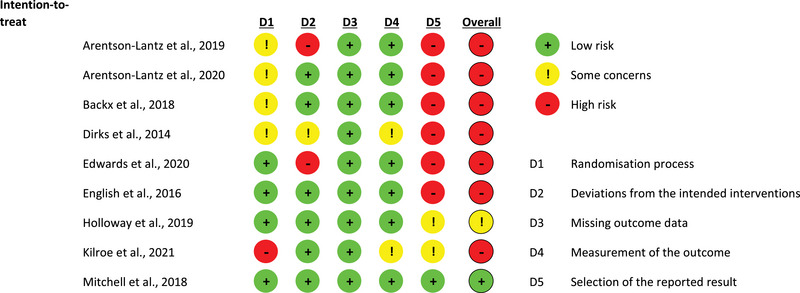
Risk‐of‐bias summary for all studies. A study is deemed high risk of bias overall if at least one domain has this result. Likewise, a study is judged to raise some concerns for risk of bias if at least one domain has this outcome. D, domain.

Only one study was considered low risk of bias in all categories. Another study showed some concerns of risk of bias, and the remaining seven studies showed a high risk of bias in at least one category (Figure 2). Selection of the reported result was the domain with the highest risk‐of‐bias score, whilst domain 3 was low risk of bias in all studies.

### 3.4 Skeletal muscle mass

All studies included in the systematic review reported assessments of skeletal muscle mass, as measured by DXA for whole‐body or leg lean mass, MRI for quadriceps muscle volume or CT scans for muscle CSA (see Table 3). Measurement periods included in the meta‐analysis were for pre‐ to post‐immobilization only, which ranged from 3 to 14 days. All studies measured muscle mass pre‐ to post‐immobilization, with one including a mid‐immobilization measurement (English et al., 2016). This time point was not included in the meta‐analysis.

**TABLE 3:** Summary of muscle mass assessments and findings from studies included in the systematic review during the immobilization period.

Reference	Muscle mass assessment	Change post‐immobilization	Back‐transformation into leg lean mass units	Significance
Arentson‐Lantz et al. (2019)	Leg lean mass (DXA)^a^	Whey: −706 ± 361 g^b^ Control: −1029 ± 503 g^b^	0.88 kg	No significant difference between groups
	Whole‐body lean mass (DXA)	Whey: −778 ± 713 g^b^ Control: −1278 ± 875 g^b^		No significant difference between groups
Arentson‐Lantz et al. (2020)	Leg lean mass (DXA)^a^	Leucine: −577 ± 212 g^b^ Control: −1038 ± 154 g^b^	1.2 kg	*P* < 0.01 vs. control
	Whole‐body lean mass (DXA)	Leucine: −1350 ± 350 g^b^ Control: −1250 ± 300 g^b^		No significant difference between groups
Backx et al. (2018)	Quadriceps CSA (CT)^a^	Leucine: 7643 ± 317 to 7164 ± 328 mm^2^ ^b^ Control: 7712 ± 324 to 7287 ± 305 mm^2^ ^b^	−0.29 kg	No significant difference between groups
	Whole‐thigh CSA (CT)	Leucine: 14,417 ± 537 mm^2^ at baseline; data for post‐immobilization not shown^b^ Control: 14,184 ± 462 mm^2^ at baseline; data for post‐immobilization not shown^b^		Data not shown
Dirks et al. (2014)	Whole‐leg muscle CSA (CT)^a^	Whey: −1.6 ± 0.6%^b^ Control: −0.7 ± 0.6%^b^	−0.46 kg	No significant difference between groups
	Quadriceps CSA (CT)	Whey: 6259 ± 259 mm^2^ pre‐immobilization vs. 6148 ± 278 mm^2^ post‐immobilization^b^ Control: 6870 ± 315 mm^2^ pre‐immobilization vs. 6778 ± 296 mm^2^ post‐immobilization^b^		No significant difference between groups
	Whole‐body lean mass (DXA)	Data not shown		No significant difference between groups
	Leg lean mass (DXA)	Data not shown		No significant difference between groups
Edwards et al. (2020)	Leg fat‐free mass (DXA)^a^	Leucine: −364 ± 113 g^b^ Control: −318 ± 68 g^b^	−0.3 kg	No significant difference between groups
English et al. (2016)	Leg lean mass (DXA)^a^	Leucine: −5.0 ± 0.8%^b^ Control: −6.8 ± 0.9%^b^	1.06 kg	No significant difference between groups
	Whole‐body lean mass (DXA)	Leucine: −2.1 ± 0.7%^b^ Control: −2.8 ± 0.6%^b^		No significant differences between groups
Holloway et al. (2019)	Quadriceps volume (MRI)^a^	Amino acid complex: −0.7 ± 1.8%^c^ Control: −2.4 ± 2.3%^c^	0.87 kg	No significant difference between groups
	Quadriceps CSA (MRI)	Amino acid complex: −0.7 ± 2.1%^c^ Control: −3.1 ± 2.1%^c^		Significantly different from control at the same time point, *P* < 0.05
Kilroe et al. (2021)	Quadriceps volume (MRI)^a^	High‐protein diet: −2.3 ± 0.4%^b^ Control: −2.0 ± 0.4%^b^	−0.24 kg	No significant difference between groups
Mitchell et al. (2018)	Leg lean mass (DXA)	Milk protein: −199 ± 256 g^b^ Control: −127 ± 256 g^b^		No significant difference between groups
	Muscle CSA at 50% femur length (pQCT)^a^	Milk protein: −682 ± 227 mm^2^ ^b^ Control: −727 ± 228 mm^2^ ^b^	0.11 kg	No significant difference between groups
	Muscle CSA at 20% femur length (pQCT)	Milk protein: −222 ± 167 mm^2^ ^b^ Control: −83 ± 195 mm^2^ ^b^		No significant difference between groups
	Muscle CSA at 66% tibia length (pQCT)	Milk protein: −346 ± 181 mm^2^ ^b^ Control: −491 ± 182 mm^2^ ^b^		No significant difference between groups

^a^ Included in meta‐analysis.

^b^ Mean ± SEM.

^c^ Mean ± SD.

Abbreviations: CSA, cross‐sectional area; DXA, dual‐energy X‐ray absorptiometry; pQCT, peripheral quantitative computed tomography.

**FIGURE 3:**
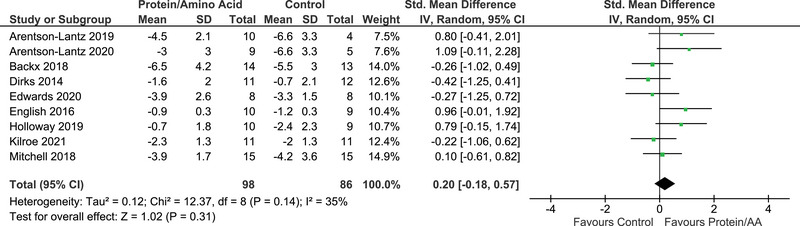
Comparison of muscle mass changes (as measured by leg lean mass, muscle cross‐sectional area or muscle volume) between the protein/amino acid group and the control group. ‘Favours control’ indicates that there was a greater loss of muscle mass (as measured by leg lean mass, muscle cross‐sectional area or muscle volume) in participants taking the protein supplementation. ‘Favours protein/AA’ indicates that there was a greater loss of muscle mass (as measured by leg lean mass, muscle cross‐sectional area or muscle volume) in participants taking the control supplementation. Abbreviation: AA, amino acid.

Percentage change from baseline to post‐immobilization was calculated from available or raw study data provided by contacted authors. Standard error was converted to standard deviation for three studies (Dirks et al., 2014; English et al., 2016; Kilroe et al., 2021). Within the meta‐analysis, methods for measuring skeletal muscle mass included leg lean/fat‐free mass via DXA (Arentson‐Lantz et al., 2020, 2019; Edwards et al., 2020; English et al., 2016), quadriceps muscle volume via MRI (Holloway et al., 2019; Kilroe et al., 2021), quadriceps CSA via CT (Backx et al., 2018) and thigh muscle CSA via CT (Dirks et al., 2014; Mitchell et al., 2018). There was no difference between protein or amino acid groups versus control in the magnitude of decline in muscle mass in response to immobilization (SMD = 0.20; 95% confidence interval: −0.18, 0.57; *Z* = 1.02; *P* = 0.31; Figure 3). A low to moderate level of heterogeneity was evident between studies (*I* ^2^ = 35%, *P* = 0.14).

Back‐transformation of the SMD into muscle volume units revealed a difference of 16.5 cm^3^ in muscle atrophy between protein or amino acid and control groups. Back‐transformation of the SMD into leg lean mass units revealed a difference of 0.22 kg in muscle atrophy between protein or amino acid and control groups. Individual study back‐transformations into leg lean mass units are presented in Table 3.

Overall, 20 different assessments of muscle mass were conducted across nine studies, and three results showed significant differences between protein or amino acid intervention and control from pre‐ to post‐immobilization (Table 3). Two studies reported a significant attenuation in the loss of leg lean mass and quadriceps CSA from pre‐ to post‐immobilization with protein or amino acid provision (Arentson‐Lantz et al., 2020; Holloway et al., 2019). Although English et al. (2016) did not report a significant difference in whole‐body lean mass between groups from pre‐ to post‐immobilization (14 days), the leucine intervention implemented did attenuate the decline in whole‐body lean mass loss at the mid‐immobilization time point (7 days) compared with control (control: −2.9 ± 0.5% vs. leucine: −1.7 ± 0.5%, group × time interaction, *P* < 0.05).

### 3.5 Muscle strength

All eligible studies included at least one measurement of muscle strength. Assessment methods included isometric torque or strength (Arentson‐Lantz et al., 2020; Edwards et al., 2020; Holloway et al., 2019; Kilroe et al., 2021; Mitchell et al., 2018), isokinetic torque (Arentson‐Lantz et al., 2020, 2019; Edwards et al., 2020; English et al., 2016; Kilroe et al., 2021), single leg extension one‐repetition maximum (Backx et al., 2018; Dirks et al., 2014) and single leg vertical jump height (Mitchell et al., 2018). Two of the nine studies reported a significant difference between control and protein or amino acid groups for measurements of strength (Table 4). Arentson‐Lantz et al. (2020) reported that the leucine supplementation group experienced a greater reduction in peak knee‐extensor torque versus a maltodextrin control, whereas English et al. (2016) reported an attenuated decline in isokinetic knee‐extensor torque at both 60°/s and 180°/s in the leucine supplementation group compared with an alanine control.

**TABLE 4:** Summary of muscle strength findings from studies included in the systematic review during the immobilization period.

Reference	Strength assessment	Change post‐immobilization	Significance
Arentson‐Lantz et al. (2019)	Isokinetic knee peak torque (60°/s)	Whey: −9.1 ± 5.5 N m^a^ Control: −16.3 ± 5.5 N m^a^	No significance between groups
Arentson‐Lantz et al. (2020)	Isometric torque (angle not specified)	Leucine: −7.1 ± 8.5 N m^a^ Control: 0.41 ± 8.5 N m^a^	No significance between groups
	Isokinetic torque (60°/s)	Leucine: −24.8 ± 4.5 N m^a^ Control: −16.3 ± 5.5 N m^a^	*P* < 0.05 between groups
Backx et al. (2018)	Single leg extension 1RM	Leucine: 63 ± 3 kg to 55 ± 2 kg pre‐ to post‐immobilization^a^ Control: 56 ± 4 kg to 53 ± 4 kg pre‐ to post‐immobilization^a^	No significance between groups (interaction effect; *P* = 0.052)
Dirks et al. (2014)	Single leg extension 1RM	Whey: 57 ± 4 kg to 51 ± 3 kg pre‐ to post‐immobilization^a^ Control: 58 ± 3 kg to 54 ± 2 kg pre‐ to post‐immobilization^a^	No significance between groups (interaction, *P* ≥ 0.05)
Edwards et al. (2020)	Isometric strength (70°)	Leucine: −224 ± 35 N^a^ Control: −165 ± 59 N^a^	No significant difference between groups (*P* = 0.852)
	Isokinetic torque (60°/s)	Leucine: −39 ± 10 N m^a^ Control: −36 ± 11 N m^a^	No significant difference between groups (*P* = 0.888)
English et al. (2016)	Isokinetic knee‐extensor torque (60°/s)	Leucine: −10 ± 4 N m^a^ Control: −24 ± 4 N m^a^	Significant interaction effect (group × time compared with pre‐bed rest, *P* < 0.05)
	Isokinetic knee‐extensor torque (180°/s)	Leucine: −5 ± 3 N m^a^ Control: −20 ± 3 N m^a^	Significant interaction effect (group × time compared with pre‐bed rest, *P* < 0.05)
Holloway et al. (2019)	Isometric knee‐extensor torque (120°)	Amino acid complex: −6.7 ± 4.7%^b^ Control: −7.3 ± 5%^b^	No significance between groups (treatment, *P* = 0.33)
Kilroe et al. (2021)	Quadriceps isometric strength (60°)	High‐protein diet: −24 ± 8%^a^ Control: −26 ± 5%^a^	No significance between groups (group × leg interaction effect, *P* > 0.05)
	Quadriceps concentric strength (60°/s)	High‐protein diet: −23 ± 8%^a^ Control: −25 ± 4%^a^	No significance between groups (group × leg interaction effect, *P* > 0.05)
	Quadriceps eccentric strength (60°/s)	High‐protein diet: −16 ± 7%^a^ Control: −23 ± 6%^a^	No significance between groups (group × leg interaction effect, *P* > 0.05)
	Hamstring isometric strength (60°)	High‐protein diet: −7 ± 2%^a^ Control: −5 ± 5%^a^	No significance between groups (group × leg interaction effect, *P* > 0.05)
	Hamstring concentric strength (60°/s)	High‐protein diet: −6.7 ± 2.8%^a^ Control: −1.1 ± 6.2%^a^	No significance between groups (group × leg interaction effect, *P* > 0.05)
	Hamstring eccentric strength (60°/s)	High‐protein diet: 3.9 ± 10%^a^ Control: −1.12 ± 6.7%^a^	No significance between groups (group × leg interaction effect, *P* > 0.05)
Mitchell et al. (2018)	Isometric knee‐extensor torque (90°)	Milk protein: −63 ± 7 N m^a^ Control: −60 ± 17 N m^a^	No significance between groups (group and group‐by‐time effects, *P* = 0.84)
	Isometric knee‐flexor torque (90°)	Milk protein: −12 ± 6 N m^a^ Control: −8 ± 6 N m^a^	No significance between groups (group and group‐by‐time effects, *P* = 0.54)
	Single leg vertical jump height	Milk protein: −4 ± 2 cm^a^ Control: −5 ± 2 cm^a^	No significance between groups (group and group‐by‐time effects, *P* = 0.26)

^a^ Mean ± SEM.

^b^ Mean ± SEM.

Abbreviation: 1RM, one‐repetition maximum.

### 3.6 Muscle protein synthesis

Four of the nine studies assessed MPS directly (Edwards et al., 2020; English et al., 2016; Kilroe et al., 2021; Mitchell et al., 2018). Methods of assessing MPS included; acute 0–4 h postprandial and postabsorptive myofibrillar protein synthesis (MyoPS) and mitochondrial protein synthesis (MitoPS) via primed constant infusion of l‐[ring‐^13^C_6_]‐phenylalanine (Edwards et al., 2020), postabsorptive and postprandial acute mixed fractional synthesis rates (FSRs) via primed constant infusion of l‐[ring‐^13^C_6_]‐phenylalanine (English et al., 2016), daily myofibrillar FSRs (MyoFSRs) via oral deuterium ingestion (Kilroe et al., 2021; Mitchell et al., 2018) and daily mitochondrial FSRs (MitoFSRs) via oral deuterium ingestion (Mitchell et al., 2018). No significant differences between groups for any measurement of MPS during the immobilization period were observed across studies (Table 5).

**TABLE 5:** Summary of muscle protein synthesis findings from studies included in the systematic review during the immobilization period.

Reference	MPS assessment	Main findings	Significance
Edwards et al. (2020)	Acute 0–4 h postprandial and postabsorptive MyoPS and MitoPS via primed constant infusion of l‐[ring‐^13^C_6_]‐phenylalanine	Change in MyoPS from postabsorptive to postprandial state in the immobilized leg Leucine: 0.006 ± 0.011%/h^a^ Control: 0.004 ± 0.004%/h^a^	No significant difference in any measurement of MyoPS or MitoPS between supplement groups (*P* > 0.05)
		Change in MitoPS from postabsorptive to postprandial state in the immobilized leg Leucine: −0.009 ± 0.02%/h^a^ Control: −0.004 ± 0.031%/h^a^	
English et al. (2016)	Postabsorptive and postprandial acute mixed FSRs via primed constant infusion of l‐[ring‐^13^C_6_]‐phenylalanine	Post‐bed rest postabsorptive FSR Leucine: 0.068 ± 0.005%/h^a^ Control: 0.042 ± 0.005%/h^a^	No significant differences between groups were detected for any MPS outcome
		Post‐bed rest postprandial FSR Leucine: 0.083 ± 0.014%/h^a^ Control: 0.090 ± 0.014%/h^a^	
Kilroe et al. (2021)	Daily MyoPS via oral deuterium ingestion	Daily MyoPS rates in the immobilized leg High‐protein diet: 1.08 ± 0.04%/day^a^ Control: 1.03 ± 0.07%/day^a^	No significant difference between groups (*P* > 0.05)
Mitchell et al. (2018)	Daily MyoPS and MitoPS via oral deuterium ingestion	Change from baseline in MyoPS rates Milk protein: −0.046 ± 0.123%/day^a^ Control: −0.123 ± 0.092%/day^a^	No significant group‐by‐time interaction (*P* = 0.150)
		Change from baseline in MitoPS rates Milk protein: −0.323 ± 0.108%/day^a^ Control: −0.431 ± 0.138%/day^a^	

^a^ Mean ± standard error.

Abbreviations: FSRs, fractional synthesis rates; MPS, muscle protein synthesis; MyoPS, myofibrillar protein synthesis; MitoPS, mitochondrial protein synthesis.

Three of the nine studies included in the systematic review assessed indirect markers of MPS (Arentson‐Lantz et al., 2020; Edwards et al., 2020; English et al., 2016), including eukaryotic translation initiation factor 4E‐binding protein 1 (4E‐BP1) (Arentson‐Lantz et al., 2020; Edwards et al., 2020; English et al., 2016), protein kinase B (AKT) (Arentson‐Lantz et al., 2020; Edwards et al., 2020), ribosomal protein S6 kinase β‐1 (S6K1) (Edwards et al., 2020; English et al., 2016), mammalian target of rapamycin (mTOR) (Arentson‐Lantz et al., 2020; English et al., 2016) and glycogen synthase kinase‐3 (GSK‐3β) (Arentson‐Lantz et al., 2020). None of the studies reported a significant difference between the protein or amino acid groups versus control in terms of the phosphorylation status of any candidate anabolic cell signalling proteins in response to the immobilization period.

## 4 DISCUSSION

### 4.1 Summary of main results

The primary aim of this systematic review and meta‐analysis was to investigate the effect of protein or amino acid‐based interventions implemented before and/or during a period of limb immobilization or bed rest on disuse‐induced changes in skeletal muscle mass in healthy young and older adults. The main finding of the meta‐analysis was no effect (SMD: 0.2; 95% confidence interval: −0.18 to 0.57, *P* = 0.31) of protein or amino acid provision in mitigating the magnitude of muscle atrophy experienced during disuse in young and older adults. Based on the small effect size (0.2), wide confidence intervals (−0.18 to 0.57) and large *P*‐value for the *Z*‐effect (*P* = 0.31), our meta‐analysis does not provide evidence to support our hypothesis that protein‐ or amino acid‐based interventions mitigate muscle disuse‐induced atrophy within the context of an ‘uncomplicated’ experimental setting. We calculated an SMD of 0.2, which indicates that the magnitude of the decline in leg lean mass following immobilization was reduced by 0.22 kg in the protein or amino acid intervention compared with the control when the SMD was back‐transformed using representative standard deviations for DXA measurements (English et al., 2016). Given that a typical error of measurement for leg lean mass, as determined by DXA, is reported to be 0.29 kg (*n* = 48, two DXA scans within 48 h; Colyer et al., 2016), this effect size lies within the standard measurement error of the predominant method (DXA) used to measure muscle mass in this systematic review, thus supporting the notion of a lack of effect of protein or amino acid provision on disuse‐induced muscle atrophy.

A secondary aim was to investigate the effect of protein or amino acid provision on muscle strength and MPS during disuse. Although a meta‐analysis was not conducted using strength and MPS datasets, based on our systematic review there was no evidence to support the use of protein or amino acid provision in mitigating the decline in strength following disuse, with only one study reporting a benefit of leucine supplementation on isokinetic knee‐extensor torque at 60°/s and 120°/s (English et al., 2016). In addition, no study reported an effect of protein or amino acid provision in stimulating MPS during a period of muscle disuse. Hence, based on currently available literature, these data indicate no clear benefit of protein or amino acid provision for preventing the disuse‐induced decline in muscle strength or MPS.

### 4.2 Agreements and disagreements with other studies or reviews

A recent systematic review and meta‐analysis of 20 intervention studies reported favourable effects of amino acid supplementation on muscle atrophy during short‐term disuse in healthy young and older adults (Ye et al., 2023). Remarkably, the calculated effect size of 0.2 (95% confidence interval: 0.09 to 0.31, *P* = 0.0007) in this previous meta‐analysis was identical to the current meta‐analysis but was statistically significant. However, in contrast to our interpretation of findings, Ye et al. (2023) concluded a beneficial effect of protein or amino acid supplementation in terms of maintaining muscle mass during disuse. Several differences in study inclusion criteria were evident between the past (Ye et al., 2023) and present systematic reviews, with Ye et al. (2023) including intervention studies that administered mixed macronutrient supplements (Fitts et al., 2007; Paddon‐Jones et al., 2005, 2004). Hence, it is difficult to delineate whether the reported favourable effect of protein or amino acid supplementation was ascribed to the ingestion of amino acids per se or whether other nutritional components of the intervention contributed to the findings. In addition, although Ye et al. (2023) relied exclusively on effect sizes to draw conclusions, it is recommended that additional transformation of datasets is conducted to aid interpretation of findings (Schünemann et al., 2023). Given that SMD is determined by the size of the effect and the variability between participants, rather than the overall mean difference between groups, SMD is presented as standard units and is more difficult to interpret (Schünemann et al., 2023). In addition, general guidance on interpreting SMDs is based on social sciences research rather than basic sciences (Cohen, 1988), thus current guidance on SMD interpretation might be considered to provide limited application to physiological outcomes. This interpretation of the effect size and inclusion of different types of nutritional intervention might explain the divergent findings between the previous (Ye et al., 2023) and present systematic review regarding the effect of protein or amino acid provision on muscle atrophy during short‐term disuse in healthy young and older adults.

The general consensus exists that the primary metabolic mechanism that underpins ‘uncomplicated’ disuse‐induced muscle atrophy relates to an impaired stimulation of MPS in response to protein feeding, termed muscle anabolic resistance (Pavis et al., 2023; Phillips & McGlory, 2014). For instance, studies investigating the effect of ingesting a meal‐like protein dose during muscle disuse have reported anabolic resistance to the intravenous infusion of amino acids (doses of 43 and 261 mg/kg/h; Glover et al., 2008) and oral ingestion of 25 g of intrinsically labelled (l‐[1‐^13^C]phenylalanine‐ and l‐[1‐^13^C]leucine) dietary protein (Wall et al., 2016) in healthy young adults. In the present systematic review, we report no effect of protein or amino acid interventions in mitigating the decline in MPS rates during short‐term muscle disuse (Edwards et al., 2020; English et al., 2016; Kilroe et al., 2021; Mitchell et al., 2018). This finding is somewhat intuitive given that the doses of ingested protein used in eligible studies ranged from 20 to 23.7 g (Dirks et al., 2014; Holloway et al., 2019; Mitchell et al., 2018) and thus were likely to be insufficient to overcome the anabolic resistance associated with muscle disuse. Accordingly, future studies are warranted to investigate the effect of higher protein dose interventions on MPS during muscle disuse.

Caution should prevail when interpreting the MPS findings from the present systematic review given the limited number of RCTs included and the observation that MPS was assessed during variable periods of muscle disuse (3−14 days) using different tracer methodologies. In this regard, 50% of studies included in this systematic review that measured MPS used an acute, intravenous infusion protocol to assess MPS outcomes (Edwards et al., 2020; English et al., 2016). Although this method offers valuable mechanistic information regarding the acute response of MPS, the timing of repeat muscle biopsies is likely to be a key factor in capturing the true response of MPS to protein provision (Witard et al., 2021). Two studies used a more contemporary deuterium oxide tracer methodology to capture integrated, free‐living MPS rates over the entire immobilization period (Kilroe et al., 2021; Mitchell et al., 2018); however, this small pool of studies reduces the chance of drawing meaningful conclusions. Hence, whilst remaining cognisant of the complex nature of correlating acute changes in MPS with chronic changes in muscle mass (Mitchell et al., 2014; Witard et al., 2021), future studies using free‐living measurements of MPS are warranted to explore more fully the impact of protein or amino acid provision on disuse‐induced changes in MPS rates.

### 4.3 Overall completeness and applicability of evidence

The wide confidence intervals calculated in the present meta‐analysis reflect the limited knowledge available regarding the effect of protein or amino acid provision on disuse‐induced muscle atrophy, which detrimentally impacts the certainty of evidence. The width of a confidence interval is determined by multiple factors, including the number of studies included in analysis, the sample size of included studies and the degree of heterogeneity between included studies (Schünemann et al., 2023). Moreover, the *P*‐value for the overall estimate of effect (*P* = 0.31) adds additional uncertainty to findings because the observed effect size is likely to be attributable to chance alone and not the intervention. Accordingly, further information is required from large‐scale trials that recruit a larger sample size before definitive conclusions can be drawn regarding the impact of protein or amino acid provision on disuse‐induced muscle atrophy.

As a note of caution, the evidence reviewed in this meta‐analysis is applicable to a population of healthy individuals who experienced short‐term (3–14 days) disuse in uncomplicated experimental conditions. Muscle atrophy in response to uncomplicated disuse is attributed primarily to an impaired response of MPS in basal, postabsorptive and postprandial conditions (Glover et al., 2008; Kilroe et al., 2020; Wall et al., 2013). However, translating these experimental findings to clinical populations is likely to be complicated by other factors known to suppress MPS and impact muscle mass, such as increased inflammation (Balage et al., 2010; Farges et al., 2002; van Hees et al., 2011) and catabolic hormones (Demling, 2009) that are upregulated during injury and diseased states. Hence, it is difficult to translate the findings of the present review directly to clinical situations that lead to muscle disuse, such as traumatic injury, burns patients and intensive care unit patients. Instead, based on the limited number of eligible studies, we contend that the evidence derived from this systematic review and meta‐analysis is not sufficient to support the recommendation to increase dietary protein intake for muscle retention within a clinical setting or muscle disuse.

### 4.4 Certainty of evidence

It is difficult to draw meaningful conclusions from studies included in this review for several reasons. First, only four of the nine studies conducted a power calculation for determination of participant sample size (Edwards et al., 2020; English et al., 2016; Kilroe et al., 2021; Mitchell et al., 2018). Of these four studies, only two were powered to detect changes in measurements of muscle mass (Edwards et al., 2020; Kilroe et al., 2021), despite eight of the nine studies stating muscle mass as their primary outcome measurement. Second, using the RoB 2 tool, we determined that 78% of eligible studies were deemed high risk of bias and included issues with randomization of studies and correct reporting of study outcomes. Third, the interventions included in this systematic review varied in terms of duration of the supplementation period (3–14 days), duration of supplementation before immobilization (0–7 days), duration and model of immobilization (3–14 days, bed rest or unilateral limb immobilization), and age of participants.

### 4.5 Potential biases in the review process

This systematic review has several strengths. First, the risk‐of‐bias assessment was undertaken in duplicate by two independent researchers (A.K.H. and T.F.), limiting potential for error attributable to conflicts of interest, lack of expertise by one author, or other author biases. Second, database searches were not limited by language or date, increasing the likelihood of capturing all relevant studies in this field. Third, the protocol was pre‐registered on PROSPERO, ensuring transparency of the review and preplanning of the protocol. Finally, the SMD was back‐transformed into meaningful units, aiding interpretation of the findings. However, several limitations also should be acknowledged. First, database searches, eligibility screening and data extraction activities were not conducted by multiple reviewers, which increases the potential for error and bias in extracted data and study selection. Second, this systematic review included a limited number of studies (*n* = 9), each of which recruited a relatively modest sample size (16–33 participants), and studies included multiple different methods for assessments of muscle mass and strength.

## 5 CONCLUSIONS

This systematic review and meta‐analysis of RCTs revealed that protein or amino acid provision before and/or during a period of ‘uncomplicated’ muscle disuse does not confer a benefit in terms of mitigating muscle atrophy. In addition, no apparent benefit of protein or amino acid provision was revealed in terms of mitigating disuse‐induced reductions in muscle strength or measurements (direct and indirect) of MPS. Given the high risk of bias and low statistical power evident in most studies included in the present systematic review, the conduct of highly powered and comprehensively reported RCTs is required before definitive conclusions can be drawn regarding the use of protein or amino acid interventions during periods of muscle disuse. Additional systematic reviews and meta‐analyses are warranted to investigate the effect of protein‐ or amino acid‐based interventions on disuse‐induced muscle atrophy within ‘complicated’ clinical settings, such as extended periods of hospitalization during and after surgery in young and older adults.

## AUTHOR CONTRIBUTIONS

Alix K. Hughes, Oliver C. Witard and Ross Pollock were responsible for the concept of the review. Alix K. Hughes and Thomas Francis conducted the risk‐of‐bias assessment. Jessica Rooney and Oliver C. Witard arbitrated disagreements in the risk‐of‐bias assessment. All authors contributed to the design and writing of the manuscript. All authors approved the final version of the manuscript and agree to be accountable for all aspects of the work in ensuring that questions related to the accuracy or integrity of any part of the work are appropriately investigated and resolved. All persons designated as authors qualify for authorship, and all those who qualify for authorship are listed.

## CONFLICT OF INTEREST

The authors declare that they have no conflicts of interest.

## Supporting information

Supporting Information
