# Supplementary material for: The effect of protein or amino acid provision on immobilization‐induced muscle atrophy in healthy adults: A systematic review and meta‐analysis
Source: Exp Physiol. 2024 Feb 29;109(6):873–88. doi: 10.1113/EP090434 (PMC11140175; doi:10.1113/EP090434)
Supplement: Supplementary file 1 — Supporting Information [file EPH-109-873-s001.docx]

## **Supporting Material: Search strategies for each database**

### **Embase**

Database: Embase Classic+Embase <1947 to 2022 December 05>

Search Strategy:

“human” filter applied

1 diet*.mp. (1211420)

2 supplement*.mp. (588095)

3 administ*.mp. (4684013)

4 provision.mp. (125707)

5 ingest*.mp. (156806)

6 1 or 2 or 3 or 4 or 5 (6168697)

7 protein.mp. (6576570)

8 amino acid*.mp. (926842)

9 Beta-hydroxy Beta-methylbut*.mp. (590)

10 7 or 8 or 9 (6916503)

11 immobili*.mp. (208438)

12 bed*rest.mp. (1685)

13 dry water immersion.mp. (11)

14 unilateral limb.mp. (401)

15 11 or 12 or 13 or 14 (210303)

16 6 and 10 (1093687)

17 15 and 16 (6664)

### **MEDLINE**

Database: Ovid MEDLINE(R) ALL <1946 to December 06, 2022>

Search Strategy:

“human” filter applied

1 diet*.mp. (860869)

2 supplement*.mp. (416999)

3 administ*.mp. (3299265)

4 provision.mp. (93407)

5 ingest*.mp. (113408)

6 1 or 2 or 3 or 4 or 5 (4306687)

7 protein.mp. (4350488)

8 amino acid*.mp. (977290)

9 beta-hydroxy beta-methylbutyrate.mp. (411)

10 7 or 8 or 9 (4726360)

11 immobili*.mp. (149987)

12 bed*rest.mp. (1079)

13 dry water immersion.mp. (9)

14 unilateral limb.mp. (272)

15 11 or 12 or 13 or 14 (151214)

16 6 and 10 (561961)

17 15 and 16 (2687)

### **Web of Science**

# Web of Science Search Strategy (v0.1)

# Database: Web of Science Core Collection

# Entitlements:

- WOS.SCI: 1900 to 2022

- WOS.AHCI: 1975 to 2022

- WOS.ESCI: 2015 to 2022

- WOS.ISTP: 1990 to 2022

- WOS.SSCI: 1956 to 2022

- WOS.ISSHP: 1990 to 2022

# Searches:

1: ALL=(diet*) Date Run: Wed Dec 07 2022 11:15:06 GMT+0000 (Greenwich Mean

Time) Results: 1167537

2: ALL=(supplement*) Date Run: Wed Dec 07 2022 11:15:22 GMT+0000 (Greenwich Mean

Time) Results: 864158

3: ALL=(administ*) Date Run: Wed Dec 07 2022 11:16:09 GMT+0000 (Greenwich Mean

Time) Results: 1932459

4: ALL=(provision) Date Run: Wed Dec 07 2022 11:16:25 GMT+0000 (Greenwich Mean

Time) Results: 280582

5: ALL=(ingest*) Date Run: Wed Dec 07 2022 11:16:35 GMT+0000 (Greenwich Mean

Time) Results: 134117

6: #5 OR #4 OR #3 OR #2 OR #1 Date Run: Wed Dec 07 2022 11:16:42 GMT+0000

(Greenwich Mean Time) Results: 4026181

7: ALL=(protein) Date Run: Wed Dec 07 2022 11:16:53 GMT+0000 (Greenwich Mean

Time) Results: 4467007

8: ALL=(amino acid*) Date Run: Wed Dec 07 2022 11:17:05 GMT+0000 (Greenwich Mean

Time) Results: 754916

9: ALL=(AA) Date Run: Wed Dec 07 2022 11:17:20 GMT+0000 (Greenwich Mean

Time) Results: 988294

10: ALL=(HMB) Date Run: Wed Dec 07 2022 11:17:27 GMT+0000 (Greenwich Mean

Time) Results: 4776

11: ALL=(Beta-hydroxy Beta-methylbut* ) Date Run: Wed Dec 07 2022 11:17:40 GMT+0000

(Greenwich Mean Time) Results: 528

12: #7 OR #8 OR #9 OR #10 OR #11 Date Run: Wed Dec 07 2022 11:18:23 GMT+0000

(Greenwich Mean Time) Results: 5750561

13: #12 AND #6 Date Run: Wed Dec 07 2022 11:18:40 GMT+0000 (Greenwich Mean

Time) Results: 574300

14: ALL=(immobili*) Date Run: Wed Dec 07 2022 11:19:04 GMT+0000 (Greenwich Mean

Time) Results: 249181

15: ALL=(bed*rest) Date Run: Wed Dec 07 2022 11:19:11 GMT+0000 (Greenwich Mean

Time) Results: 901

16: ALL=(dry water immersion) Date Run: Wed Dec 07 2022 11:19:20 GMT+0000

(Greenwich Mean Time) Results: 2606

17: ALL=(unilateral limb) Date Run: Wed Dec 07 2022 11:19:38 GMT+0000 (Greenwich

Mean Time) Results: 8553

18: #17 OR #16 OR #15 OR #14 Date Run: Wed Dec 07 2022 11:19:48 GMT+0000

(Greenwich Mean Time) Results: 260923

19: #18 AND #13 Date Run: Wed Dec 07 2022 11:21:59 GMT+0000 (Greenwich Mean

Time) Results: 3455

20: #18 AND #13 Date Run: Wed Dec 07 2022 11:22:03 GMT+0000 (Greenwich Mean

Time) Results: 3455

### **PubMed**

| Search number | Query | Sort By | Filters | Search Details | Results | Time |
| --- | --- | --- | --- | --- | --- | --- |
| 21 | (#13) AND (#20) | First Author | Humans | ((("diet*"[All Fields] AND "humans"[MeSH Terms]) OR ("supplement*"[All Fields] AND "humans"[MeSH Terms]) OR ("administ*"[All Fields] AND "humans"[MeSH Terms]) OR (("provision"[All Fields] OR "provisioned"[All Fields] OR "provisioning"[All Fields] OR "provisions"[All Fields]) AND "humans"[MeSH Terms]) OR ("ingest*"[All Fields] AND "humans"[MeSH Terms])) AND "humans"[MeSH Terms] AND (((("protein s"[All Fields] OR "proteinous"[All Fields] OR "proteins"[MeSH Terms] OR "proteins"[All Fields] OR "protein"[All Fields]) AND "humans"[MeSH Terms]) OR (("amino"[All Fields] OR "aminos"[All Fields]) AND "acid*"[All Fields] AND "humans"[MeSH Terms]) OR (("analogs and derivatives"[MeSH Subheading] OR ("analogs"[All Fields] AND "derivatives"[All Fields]) OR "analogs and derivatives"[All Fields] OR "aa"[All Fields]) AND "humans"[MeSH Terms]) OR ("HMB"[All Fields] AND "humans"[MeSH Terms]) OR ("Beta-hydroxy"[All Fields] AND "beta methylbut*"[All Fields] AND "humans"[MeSH Terms])) AND "humans"[MeSH Terms]) AND "humans"[MeSH Terms] AND ((("immobili*"[All Fields] AND "humans"[MeSH Terms]) OR (("bed rest"[MeSH Terms] OR ("bed"[All Fields] AND "rest"[All Fields]) OR "bed rest"[All Fields]) AND "humans"[MeSH Terms]) OR (("bed rest"[MeSH Terms] OR ("bed"[All Fields] AND "rest"[All Fields]) OR "bed rest"[All Fields] OR "bedrest"[All Fields]) AND "humans"[MeSH Terms]) OR (("bed rest"[MeSH Terms] OR ("bed"[All Fields] AND "rest"[All Fields]) OR "bed rest"[All Fields]) AND "humans"[MeSH Terms]) OR ("dry"[All Fields] AND ("water"[MeSH Terms] OR "water"[All Fields] OR "watering"[All Fields] OR "water s"[All Fields] OR "watered"[All Fields] OR "waterer"[All Fields] OR "waterers"[All Fields] OR "waterings"[All Fields] OR "waters"[All Fields]) AND ("immerse"[All Fields] OR "immersed"[All Fields] OR "immerses"[All Fields] OR "immersing"[All Fields] OR "immersion"[MeSH Terms] OR "immersion"[All Fields] OR "immersions"[All Fields] OR "immersive"[All Fields] OR "immersiveness"[All Fields]) AND "humans"[MeSH Terms]) OR (("unilateral"[All Fields] OR "unilaterally"[All Fields] OR "unilaterals"[All Fields]) AND ("extremities"[MeSH Terms] OR "extremities"[All Fields] OR "limb"[All Fields]) AND "humans"[MeSH Terms])) AND "humans"[MeSH Terms])) AND (humans[Filter]) | 2,531 | 11:38:36 |
| 20 | (((((#14) OR (#15)) OR (#16)) OR (#17)) OR (#18)) OR (#19) | First Author | Humans | (("immobili*"[All Fields] AND "humans"[MeSH Terms]) OR (("bed rest"[MeSH Terms] OR ("bed"[All Fields] AND "rest"[All Fields]) OR "bed rest"[All Fields]) AND "humans"[MeSH Terms]) OR (("bed rest"[MeSH Terms] OR ("bed"[All Fields] AND "rest"[All Fields]) OR "bed rest"[All Fields] OR "bedrest"[All Fields]) AND "humans"[MeSH Terms]) OR (("bed rest"[MeSH Terms] OR ("bed"[All Fields] AND "rest"[All Fields]) OR "bed rest"[All Fields]) AND "humans"[MeSH Terms]) OR ("dry"[All Fields] AND ("water"[MeSH Terms] OR "water"[All Fields] OR "watering"[All Fields] OR "water s"[All Fields] OR "watered"[All Fields] OR "waterer"[All Fields] OR "waterers"[All Fields] OR "waterings"[All Fields] OR "waters"[All Fields]) AND ("immerse"[All Fields] OR "immersed"[All Fields] OR "immerses"[All Fields] OR "immersing"[All Fields] OR "immersion"[MeSH Terms] OR "immersion"[All Fields] OR "immersions"[All Fields] OR "immersive"[All Fields] OR "immersiveness"[All Fields]) AND "humans"[MeSH Terms]) OR (("unilateral"[All Fields] OR "unilaterally"[All Fields] OR "unilaterals"[All Fields]) AND ("extremities"[MeSH Terms] OR "extremities"[All Fields] OR "limb"[All Fields]) AND "humans"[MeSH Terms])) AND (humans[Filter]) | 73,326 | 05:36:23 |
| 19 | unilateral limb | First Author | Humans | (("unilateral"[All Fields] OR "unilaterally"[All Fields] OR "unilaterals"[All Fields]) AND ("extremities"[MeSH Terms] OR "extremities"[All Fields] OR "limb"[All Fields])) AND (humans[Filter]) | 10,493 | 05:35:10 |
| 18 | dry water immersion | First Author | Humans | ("dry"[All Fields] AND ("water"[MeSH Terms] OR "water"[All Fields] OR "watering"[All Fields] OR "water s"[All Fields] OR "watered"[All Fields] OR "waterer"[All Fields] OR "waterers"[All Fields] OR "waterings"[All Fields] OR "waters"[All Fields]) AND ("immerse"[All Fields] OR "immersed"[All Fields] OR "immerses"[All Fields] OR "immersing"[All Fields] OR "immersion"[MeSH Terms] OR "immersion"[All Fields] OR "immersions"[All Fields] OR "immersive"[All Fields] OR "immersiveness"[All Fields])) AND (humans[Filter]) | 333 | 05:35:03 |
| 17 | bed-rest | First Author | Humans | ("bed rest"[MeSH Terms] OR ("bed"[All Fields] AND "rest"[All Fields]) OR "bed rest"[All Fields]) AND (humans[Filter]) | 7,904 | 05:34:05 |
| 16 | bedrest | First Author | Humans | ("bed rest"[MeSH Terms] OR ("bed"[All Fields] AND "rest"[All Fields]) OR "bed rest"[All Fields] OR "bedrest"[All Fields]) AND (humans[Filter]) | 8,480 | 05:33:59 |
| 15 | bed rest | First Author | Humans | ("bed rest"[MeSH Terms] OR ("bed"[All Fields] AND "rest"[All Fields]) OR "bed rest"[All Fields]) AND (humans[Filter]) | 7,904 | 05:33:52 |
| 14 | immobili* | First Author | Humans | ("immobili*"[All Fields]) AND (humans[Filter]) | 54,907 | 05:33:02 |
| 13 | (#6) AND (#12) | First Author | Humans | ((("diet*"[All Fields] AND "humans"[MeSH Terms]) OR ("supplement*"[All Fields] AND "humans"[MeSH Terms]) OR ("administ*"[All Fields] AND "humans"[MeSH Terms]) OR (("provision"[All Fields] OR "provisioned"[All Fields] OR "provisioning"[All Fields] OR "provisions"[All Fields]) AND "humans"[MeSH Terms]) OR ("ingest*"[All Fields] AND "humans"[MeSH Terms])) AND "humans"[MeSH Terms] AND (((("protein s"[All Fields] OR "proteinous"[All Fields] OR "proteins"[MeSH Terms] OR "proteins"[All Fields] OR "protein"[All Fields]) AND "humans"[MeSH Terms]) OR (("amino"[All Fields] OR "aminos"[All Fields]) AND "acid*"[All Fields] AND "humans"[MeSH Terms]) OR (("analogs and derivatives"[MeSH Subheading] OR ("analogs"[All Fields] AND "derivatives"[All Fields]) OR "analogs and derivatives"[All Fields] OR "aa"[All Fields]) AND "humans"[MeSH Terms]) OR ("HMB"[All Fields] AND "humans"[MeSH Terms]) OR ("Beta-hydroxy"[All Fields] AND "beta methylbut*"[All Fields] AND "humans"[MeSH Terms])) AND "humans"[MeSH Terms])) AND (humans[Filter]) | 736,030 | 05:32:34 |
| 12 | ((((#7) OR (#8)) OR (#9)) OR (#10)) OR (#11) | First Author | Humans | ((("protein s"[All Fields] OR "proteinous"[All Fields] OR "proteins"[MeSH Terms] OR "proteins"[All Fields] OR "protein"[All Fields]) AND "humans"[MeSH Terms]) OR (("amino"[All Fields] OR "aminos"[All Fields]) AND "acid*"[All Fields] AND "humans"[MeSH Terms]) OR (("analogs and derivatives"[MeSH Subheading] OR ("analogs"[All Fields] AND "derivatives"[All Fields]) OR "analogs and derivatives"[All Fields] OR "aa"[All Fields]) AND "humans"[MeSH Terms]) OR ("HMB"[All Fields] AND "humans"[MeSH Terms]) OR ("Beta-hydroxy"[All Fields] AND "beta methylbut*"[All Fields] AND "humans"[MeSH Terms])) AND (humans[Filter]) | 4,804,548 | 05:32:06 |
| 11 | Beta-hydroxy Beta-methylbut* | First Author | Humans | ("Beta-hydroxy"[All Fields] AND "beta methylbut*"[All Fields]) AND (humans[Filter]) | 268 | 05:31:06 |
| 10 | HMB | First Author | Humans | ("HMB"[All Fields]) AND (humans[Filter]) | 2,512 | 05:30:42 |
| 9 | AA | First Author | Humans | ("analogs and derivatives"[MeSH Subheading] OR ("analogs"[All Fields] AND "derivatives"[All Fields]) OR "analogs and derivatives"[All Fields] OR "aa"[All Fields]) AND (humans[Filter]) | 483,856 | 05:29:57 |
| 8 | amino acid* | First Author | Humans | (("amino"[All Fields] OR "aminos"[All Fields]) AND "acid*"[All Fields]) AND (humans[Filter]) | 385,166 | 05:29:51 |
| 7 | protein | First Author | Humans | ("protein s"[All Fields] OR "proteinous"[All Fields] OR "proteins"[MeSH Terms] OR "proteins"[All Fields] OR "protein"[All Fields]) AND (humans[Filter]) | 4,468,447 | 05:29:40 |
| 6 | ((((#1) OR (#2)) OR (#3)) OR (#4)) OR (#5) | First Author | Humans | (("diet*"[All Fields] AND "humans"[MeSH Terms]) OR ("supplement*"[All Fields] AND "humans"[MeSH Terms]) OR ("administ*"[All Fields] AND "humans"[MeSH Terms]) OR (("provision"[All Fields] OR "provisioned"[All Fields] OR "provisioning"[All Fields] OR "provisions"[All Fields]) AND "humans"[MeSH Terms]) OR ("ingest*"[All Fields] AND "humans"[MeSH Terms])) AND (humans[Filter]) | 2,923,346 | 05:29:12 |
| 5 | ingest* | First Author | Humans | ("ingest*"[All Fields]) AND (humans[Filter]) | 70,907 | 05:27:45 |
| 4 | provision | First Author | Humans | ("provision"[All Fields] OR "provisioned"[All Fields] OR "provisioning"[All Fields] OR "provisions"[All Fields]) AND (humans[Filter]) | 79,349 | 05:27:35 |
| 3 | administ* | First Author | Humans | ("administ*"[All Fields]) AND (humans[Filter]) | 2,313,603 | 05:22:01 |
| 2 | supplement* | First Author | Humans | ("supplement*"[All Fields]) AND (humans[Filter]) | 233,006 | 05:21:48 |
| 1 | diet* | First Author | Humans | ("diet*"[All Fields]) AND (humans[Filter]) | 518,761 | 05:21:10 |
| 0 | Clipboard |  |  | 29722847,17070743,15447920,15691900,12053941,11542811,17509313,33471625,23079398,11543081,9920085,11541183,8042526,14008124,11538781,15486040,32303743,26578714,24108315,26402815,36056473,17655736,18094071,17641219,19368813,26173027,2203254,24184274,12471043,11200976,10467608,31907525,28705993,32164702,9768669,19232702,19915502,10578019,19448702,29266264,32470312,28482746,32215553,23725606,16984982,15987870,15598679,15356032,32324477,18758029,22455386,32918768,19196916,33693737,34081111,22995395,29122965,19059391,10902934,8304043,10444434,29077223,19229964,1877520,24458754,7626318,18948558,32469388,18372698,32609523,22595648,34632796,12197542,11543485,18372696,28177714,15900645,11541179,31046520,1509887,1601796,18372700,32651634,18955382,2435445,31461862,35477971,21596917,17409123,19419806,8928769,26718415,32910813,17928515,18372697,22338078,36048851,30645176,29656554,31721213,24919692,23514626,27395413,29377592,19476168,19470803,28250244,18483167,25317071,20928906,32130485,27516541,25524969,16568340,33630674,27742138,18842781,17684207,15131238,21723970,35805205,19765953,12909597,29772844,33831949,21812030,32191600,30689727,18372693,18202168,18936221[UID] | 131 | 11:43:54 |

### **Clinical Trials**

ClinicalTrials.gov

Searched on 13/12/2022 with the term “immobilisation” returning 695 results
